# Diabetes mellitus and inequalities in the equipment and use of information technologies as a socioeconomic determinant of health in Spain

**DOI:** 10.3389/fpubh.2022.1033461

**Published:** 2023-01-09

**Authors:** Irene Bosch-Frigola, Fernando Coca-Villalba, María Jose Pérez-Lacasta, Misericordia Carles-Lavila

**Affiliations:** ^1^Department of Economics, Rovira i Virgili University, Reus, Spain; ^2^Facultad de Comunicación y Ciencias Sociales, Universidad San Jorge, Zaragoza, Spain; ^3^Research Group on Statistics, Economic Evaluation and Health (GRAEES), Reus, Spain; ^4^Research Center on Economics and Sustainability (ECO-SOS), Reus, Spain

**Keywords:** diabetes mellitus, factor analysis of mixed data, social determinants of health, economics, inequalities

## Abstract

Inequalities in the equipment and use of information and communications technology (ICT) in Spanish households can lead to users being unable to access certain information or to carry out certain procedures. Accessibility to ICT is considered a social determinant of health (SDOH) because it can generate inequalities in access to information and in managing access to health services. In the face of a chronic illness such as diabetes mellitus (DM)—for which a comprehensive approach is complex and its complications have a direct impact on current healthcare systems—all the resources that patients may have are welcome. We aimed to analyze hospitalizations and amputations as direct consequences of DM among the autonomous communities of Spain (ACS) in 2019, along with socioeconomic factors related to health, including inequalities in access to ICT between territories, as well as citizens' interest in online information searches about DM. We used different databases such as that of the Ministerio de Sanidad (Spain's health ministry), Ministerio de Asuntos Económicos y transformación (Ministry of Economic Affairs and Digital Transformation), Google Trends (GT), and the Instituto Nacional de Estadística (Spain's national institute of statistics). We examined the data with R software. We employed a geolocation approach and performed multivariate analysis (specifically factor analysis of mixed data [FAMD]) to evaluate the aggregate interest in health information related to DM in different regions of Spain grounded in online search behavior. The use of FAMD allowed us to adjust the techniques of principal component analysis (PCA) and multiple correspondence analysis (MCA) to detect differences between the direct consequences of DM, citizen's interest in this non-communicable disease, and socioeconomic factors and inequalities in access to ICT in aggregate form between the country's different ACS. The results show how SDOH, such as poverty and education level, are related to the ACS with the highest number of homes that cite the cost of connection or equipment as the reason for not having ICT at home. These regions also have a greater number of hospitalizations due to DM. Given that in Spain, there are certain differences in accessibility in terms of the cost to households, in the case of DM, we take this issue into account from the standpoint of an integral approach by health policies.

## Introduction

### The equipment and use of information and communications technology as a social determinant of health

The social determinants of health (SDOH) are essential for achieving good public health. They are framed in an environment where political, social, and economic forces interact and where people are born, grow, live, work, and age. Surveillance in the field of public health is therefore crucial. Surveillance encompasses not only the systematic, continuous collection of data on the population's health; information analysis and interpretation are also critical to plan, implement, and evaluate public health actions (1–4). According to (5), both digital literacy and Internet connectivity can be understood as “super [SDOH]” because they include all other social determinants related to health. In this regard, there are remarkable inequalities in the world. According to (6), between 2005 and 2019, the number of internet users grew by 10% on average. In 2019, the global penetration rate increased over 53%, and Europe had the highest internet usage rates. In 2019, the vast majority of the world's population lived within reach of a mobile network and, in developed countries, about 87% of the population used the internet.

Organisation for Economic Co-operation and Development OECD, (7) stresses that access to and use of information and communications technology (ICT) translates into real social benefits. In OECD countries, the population that has come to use the internet has grown by 30 percentage points over the last decade. In most member states, almost all young people between the ages of 16 and 24 use the internet on a daily basis. However, among individuals between 55 and 74 years old, the median stands at 55%, with very considerable differences depending on the country analyzed (8).

The use of ICT by all people—including fundamental human rights such as privacy and ensuring the ethical use of data—must prevail. Although there may be a risk of fostering inequality and prejudice between groups who have access to data and know how to use them, and those who do not, the discrepancies that may arise and that could end up hindering access to ICT must be avoided (9). Although exclusion related to the information society can take place passively (due to social and cultural environmental conditions) or actively (for reasons external to the individual, whether governmental or political), some authors consider that although access to digital resources is more costly for people without incomes, when they have access to ICT and are going to use it, they may be more interested in it. Hence, not having access to ICT does not imply social exclusion, but not having ICT means that the situation of exclusion is exacerbated for the population without access to it. Given this scenario, it is vital to highlight the role of the “digital divide” and to distinguish between the impact of “access” and “use” of ICT (10, 11).

### Health care services and digital health technologies

The emergence of electronic health (eHealth) marks a breakthrough as it represents support for citizens' health related to (among other aspects) healthcare services, education, and surveillance (12). Currently, eHealth is implemented in different governments as well as public administrations, and embodies one more step toward so-called electronic government, since the use of eHealth, in some countries, denotes support for universal health coverage (13–15).

While digital health technologies aim to promote the efficiency of healthcare delivery by offering better medical services to citizens (both in the public and private sectors), their application might not always be homogeneous (16, 17).

Health informatics interventions are designed to improve the quality of health and the safety of healthcare, even though they might lead to inequalities and might not benefit the most disadvantaged people (18). There may be a relationship between an individual's social status, the probability of contracting a disease, and his/her life expectancy; in this context, social inequalities could end up affecting people (19). Faced with this reality, the citizen's experience is essential in terms of patient-centered health care delivery and involvement (20).

### Diabetes mellitus in the equipment and use of ICT

Diabetes mellitus (DM) is a serious chronic disease (CD) that is on the rise (21–23). Regardless of the patient's age, good care in the context of this CD is not easy, and has to be approached with the help of ongoing medical care and multifactorial interventions that do not simply focus on glycemic control (24, 25). To achieve this, there is a need to reduce (as much as possible) the risk of developing complications associated with DM through good self-management (26). Additional factors to consider include a proper diet, supervised physical exercise, healthy behaviors, and control of indicators such as blood pressure, lipid values, and thrombotic control (27, 28). A lot of sacrifice is required and will only be attained if the patient has a lot of discipline, support from his/her environment, and seeks help from professionals to establish guidelines to avoid dreaded consequences, such as the need to be hospitalized due to diabetic decompensation, or limb amputations with all the emotional and economic implications that they entail (29–34).

Ongoing digitization in DM management offers new opportunities for patients, their environment, and healthcare professionals. Improvements have had a particular impact on patients' glycemic control, as well as enhancing patient autonomy and quality of life (35). The (36) uses the term “diabetes technology” to describe the hardware, devices, and software that people use to manage DM, ranging from controlling blood glucose levels to lifestyle. Previously, DM technology consisted of insulin administered by various devices, as well as monitoring using a meter or a continuous glucose monitor. Currently, hybrid devices have been available that not only monitor glucose; they can also administer insulin, and some of them have built-in software that assists the patient and others in his/her environment in terms of diabetic control (22, 37).

Thus, awakening interest and encouraging the involvement of patients (young and old) and others in their environment will raise awareness of this reality. The technology developed in recent decades can be of great help to patients, others in their environment, and health professionals in order to provide patients with resources to face their CD (38–40). New innovations include the Móvil electronic device (MED) (41–50).

Special emphasis should also be placed on the fact that on the internet, users can exchange information with other people, as well as discuss any queries they may have in relation to DM. To this end, users must have the appropriate technological equipment (e.g., suitable hardware and software, an internet connection, etc.). Since the most widely used search engine is Google, it is possible to use Google Trends (GT) to monitor what the population can find out about DM through this search engine (51).

### Diabetes mellitus and the impact of digital competency on healthcare in Spain

As discussed above, accessibility to ICT is considered an SDOH; inequality in access to information for the population, as well as managing access to health services, are critical. Given the need to establish digital governance, digital competence is one of the factors that can have the greatest impact on social inequality (52).

Furthermore, the importance of the educational structure in explaining proper use of the internet should be emphasized (53). According to (54), studies carried out in Spain on the determinants of the use of e-Government found that digital skills and confidence in the internet could have an impact on the use of e-Government. This also suggests that digital skills are affected by citizens' resources, economic and education level, age, and gender. The same study noted that trust in the internet was conditioned (among other factors) by the concern of being the object of advertising. Extrapolating these findings to health issues in Spain, within the context of ongoing improvement, the Quality Plan for the National Health System of Spain (2006–2010) included issues such as protection, health promotion, and prevention. These actions are in line with the principles established in the Tallinn Charter, signed in 2008 by the Spanish government. Within this framework, we carried out the present study to reduce health inequality. The abovementioned actions are framed within the general policies of Spain's Ministry of Health and Social Policy, and one of those actions relies on ICT to improve care provided to the Spanish population (55).

From the standpoint of worrisome data related to inequality, death, prevalence, and cost in Spain (56), a CD such as DM must be seriously considered. Previous referrals to the need for patient care must always be present, because poor care related to DM can have serious consequences for the patient's health, as well as for economic costs (56–61). Unfortunately, two of the sequelae of this poorly controlled illness are hospitalizations (62–64) and amputations (65–68).

We aimed to analyze the impact of inadequate diabetic control as direct consequences of DM among the autonomous communities of Spain (ACS), along with socioeconomic factors related to health, including inequalities in access to ICT as an SDOH (between territories, as well as citizens' interest in online information searches about DM. Hence, we aimed to scrutinize hospitalizations and amputations as direct consequences of DM in ACS, as well as their relationship with socioeconomic factors of health, including inequality in access to ICT throughout the ACS and the population's behavior toward online information searches about DM in 2019 (before pre-pandemic trends).

This research paper is structured as follows: Section 2 “materials and methods” explains the steps to set up the database as well as the methodology and software used. Section 3 “Results” shows the results of the research. Section 4 “Discussion” shows the relevant findings of the present work.

## Materials and methods

We performed our study in the following steps:

**(1) Database design and sources searched**

In order to build a database, we needed to check different sources.

(a) **Government sources**

(a1) Spain health ministry (69).

- *A profile of the Spanish population: Education level and poverty*. We coded these variables as “education” and “poverty.”
- *Health data related to hospitalizations and amputations caused by DM*. We coded these variables as “hospital” and “amputation.”
- *Public health spending managed by the ACS per inhabitant*. We coded this variable as “healthcare.”

(a2) Ministry of Economic Affairs and Digital Transformation (State Secretariat for Digitalization and Artificial Intelligence Department) (70). We extracted information related to the percentage of broadband coverage through different connection technologies and speeds (Table 1).

- *Asymmetric Digital Subscriber Line (“ADSL* ≥ *2 Mbps” and “ADSL* ≥ *10 Mbps”)*. We coded these variables as “adsl.2 mb” and “adsl.10 mb.”
- *Very High-Rate Digital Subscriber Line (VDSL)*. We coded this variable as “vdsl.”
- *Hybrid Fiber Coaxial (HFC)*. We coded this variable as “hfc.”
- *Fiber to the Home (FTTH)*. We coded this variable as “ftth.”
- *Wireless* ≥ *30 Mbps*. We coded this variable as “in.30.”
- *Universal Mobile Telecommunications System (UMTS) with High-Speed Packet Access (HSPA)*. “umts.”
- *Long-Term Evolution (LTE) 4G*. We coded this variable as “lte.”
- *Speech per coverage* ≥*30 Mbps*. We coded this variable as “sp30 mb.”
- *Speech per coverage* ≥*100 Mbps*. We coded this variable as “sp100 mb.”
- *Number of households*. We extracted this information from (71) since it contains data on the number of households for 2019.

**Table 1:** Descriptive summary of the variables of broadband technology coverage in Spain in 2019.

	**adsl.2mb**	**adsl.10mb**	**vdsl**	**hfc**	**ftth**	**in.30**	**umts**	**lte**	**sp30mb**	**spl00mb**
Mean	0,88171	0,71159	0,12274	0,5103	0,7606	0,3704	0,998776	0,996471	0,94055	0,80481
Variance	0,00420	0,00586	0,00056	0,0377	0,0132	0,0521	0,000003	0,000015	0,00092	0,00897
Standard deviation	0,06481	0,07654	0,02369	0,1941	0,1151	0,2282	0,001642	0,003855	0,03034	0,09471
Skewness	−1,5479	−0,9029	0,7205	−0,3798	−0,3299	0,6439	−3,0127	−1,5979	0,0389	−0,1188
Kurtosis	5,7595	3,8053	3,0353	3,7106	3,3878	3,3008	13,4080	5,0310	2,0319	2,7320
Median	0,89190	0,73450	0,12000	0,5337	0,7814	0,3915	0,999400	0,998600	0,94190	0,81160
Minimun	0,70020	0,52710	0,09040	0,0660	0,5030	0,0300	0,993000	0,986500	0,89600	0,64120
Maximun	0,95820	0,82180	0,17670	0,8870	0,9729	0,8323	1,000000	0,999900	0,98910	0,97300
Range	0,25800	0,29470	0,08630	0,8210	0,4699	0,8023	0,007000	0,013400	0,09310	0,33180
Count	17	17	17	17	17	17	17	17	17	17
1st cuartil	0,86400	0,67340	0,10580	0,4239	0,6888	0,1834	0,998400	0,995700	0,91450	0,75550
3rd cuartil	0,92530	0,75790	0,13320	0,6189	0,8113	0,4235	0,999700	0,999100	0,96150	0,85620
Interquartile range	0,06130	0,08450	0,02740	0,1950	0,1225	0,2401	0,001300	0,003400	0,04700	0,10070

Source: Ministry of economic affairs and digital transformation (state secretariat for digitalization and artificial intelligence department).

(b) **Instituto Nacional de Estad**í**stica** **(****71****)**

Using the data provided by Instituto Nacional de Estadística, it was feasible to set up the database based on the information related to these four areas:

(b1) *Area of ACS per square kilometer*. We coded this variable as “housing_density.”
  We extracted the information in sections b2, b3, and b4 from the “Survey on Equipment and Use of [ICT] in Households, 2019.” The population analyzed was between 16 and 74 years of age.
(b2) *ICT product equipment in homes*.
  *The internet access of primary households by ACS and type of connection*. We considered the number of households and those with broadband and narrowband connections. We coded these variables as “broadband” and “narrowband.”
(b3) *Internet services used for specific reasons according to the type of service and by ACS*. This relates to citizens who searched for information about health issues. We coded this variable as “health.info.pop.”
(b4) *The main reasons for which main homes do not have internet access by ACS*.
  Reasons included the cost of having hardware (we coded this variable as “hardware.cost”) or the cost of the connection (we coded this variable as “connect.cost”).

(c) **Google Trends**

The internet is a good environment for understanding individuals' concerns and needs. GT is a very good tool to monitor citizens' interests, parameterizing the information collected through the search engine's users (51). In this way, GT collected data in 2019 through users who searched for the term “diabetes mellitus,” which shaped the respective proportion through the relative search volume (RSV). The information obtained by the GT has been used as a subrogate of online health information seeking behavior. We parameterized the respective normalization from 0 to 100 and matched it to the highest proportion of the searched term. We coded this variable as “hits.dm.”

**(2) Methodology and software**

(a) **Methodology**

To identify patterns to carry out the current research, it became necessary to set up the database, relying on the sources indicated above. This involved the following steps:

(a1) We created the corresponding projection on different maps of Spain (see Appendix 1).
(a2) We used a multivariate method to treat the variables. We took into account that the variables extracted were both quantitative and qualitative. As such, we used factorial analysis of mixed data (FAMD) to classify the data (72, 73).

(b) **Software**

We used several free R software libraries to achieve the abovementioned outputs (72–74). We used FactoMineR package and FactoExtra package for FAMD. To create the maps included in this article and in the appendices (Appendix 1 and **Figure 4**), we employed several libraries, including the tmaptools package, the maptools package, the tmap package, the rgdal package, the tidyverse package, the sf package, the raster package, the rworldxtra package, the leaflet package, and the spdep package.

## Results

We had to find the density of households in each ACS (“housing_density”). This variable is obtained by dividing the number of households (ACS) by the surface area (in km^2^) (ACS).

From these data, we extracted the quartile corresponding to this ratio for the ACS among the Spanish regions analyzed. This variable represents the quartile that denotes the density of the number of households between surface area (in km^2^) in relation to Spain as a whole. The “housing_density” variable characterizes each region according to the population of households and its dispersion in the region as an approximation of the pressure of the demand for technological coverage existing in each ACS.

We obtained a variable from the broadband coverage data. This qualitative variable represents the group to which the ACS belongs according to the percentage of broadband technology implementation and coverage speed in the region. Note that we coded this variable as “TICR.” Please, be aware that it refers to the existing technological coverage in 2019 for each of the ACS: *ADSL* ≥ *2 Mbp, ADSL* ≥ *10 Mbps, VDSL, HFC, FTTH, wireless* ≥ *30 Mbps, UMTS with HSPA, LTE, sp30 mb and sp100 mb*.

Using FAMD, we computed a qualitative variable (TICR) that described five possible clusters to which an autonomous region belonged according to the characteristics studied. The results generated by applying FAMD to obtain the TICR variable are shown below.

Supplementary Figure S1 (see Appendix 2) presents the sedimentation graph with the five dimensions, into which we grouped the variables of broadband technology coverage and the existing connection speed in each ACS (see Table 2).

**Table 2:** Percentage of inertia explained by each FAMD dimensions.

	**Eigenvalue**	**Variance percent**	**Cumulative variance percent**
Dim.1	4,177596	32,135357	32,135357
Dim.2	2,981351	22,933469	55,068826
Dim.3	1,976681	15,205236	70,274062
Dim.4	1,423336	10,948736	81,222798
Dim.5	1,042255	8,017348	89,240146

Supplementary Figures S2–S7 (see Appendix 2) outline the variables grouped according to their contribution to each dimension.

Table 3 displays the outcomes of the contributions of the variables analyzed in each dimension. Dimension 1 groups coverage by LTE broadband technologies and connection speed above 100 Mbps (Supplementary Figure S2, see Appendix 2). Dimension 2 groups broadband coverage technologies by ADSL ≥10 Mbps, ADSL ≥ 2 Mbps, VDSL, and FTTH (Supplementary Figure S3, see Appendix 2).

**Table 3:** Contributions of technology–related variables.

	**Dim.1**	**Dim.2**	**Dim.3**	**Dim.4**	**Dim.5**
adsl.2mb	0,031	**29,113**	1,243	3,270	0,133
adsl.10mb	2,460	**24,360**	0,342	4,724	4,099
vdsl	7,170	**10,897**	6,145	0,882	9,358
hfc	6,773	0,592	**16,346**	11,190	11,436
ftth	9,351	**16,047**	0,421	1,003	0,454
in.30	2,211	0,211	18,271	**19,879**	1,133
umts	12,756	O,000	0,232	**22,766**	4,907
lte	**14,693**	1,436	2,555	11,827	5,758
sp30mb	6,802	2,150	**24,492**	2,326	0,102
Sp100mb	**17,057**	7,426	0,407	0,439	1,704
Housing.density	20,694	7,766	29,544	21,693	**60,916**

Values in bold show those that are higher.

Dimension 3 includes HFC broadband coverage technologies and connection speeds ≥30 Mbps (Supplementary Figure S4, see Appendix 2). Dimension 4 groups wireless technologies >30 Mbps and UMTS with HSPA (Supplementary Figure S5, see Appendix 2).

Lastly, Dimension 5 is reserved for the qualitative variable “housing_density.”

Supplementary Figure S6 (see Appendix 2) shows the data obtained for the density of households per km^2^ in each ACS, and the map factor of the distribution of this variable, “housing_density,” as a qualitative variable. Correlation circle of the quantitative variables is shown in Supplementary Figure S7 (see Appendix 2).

**Figure 1:**
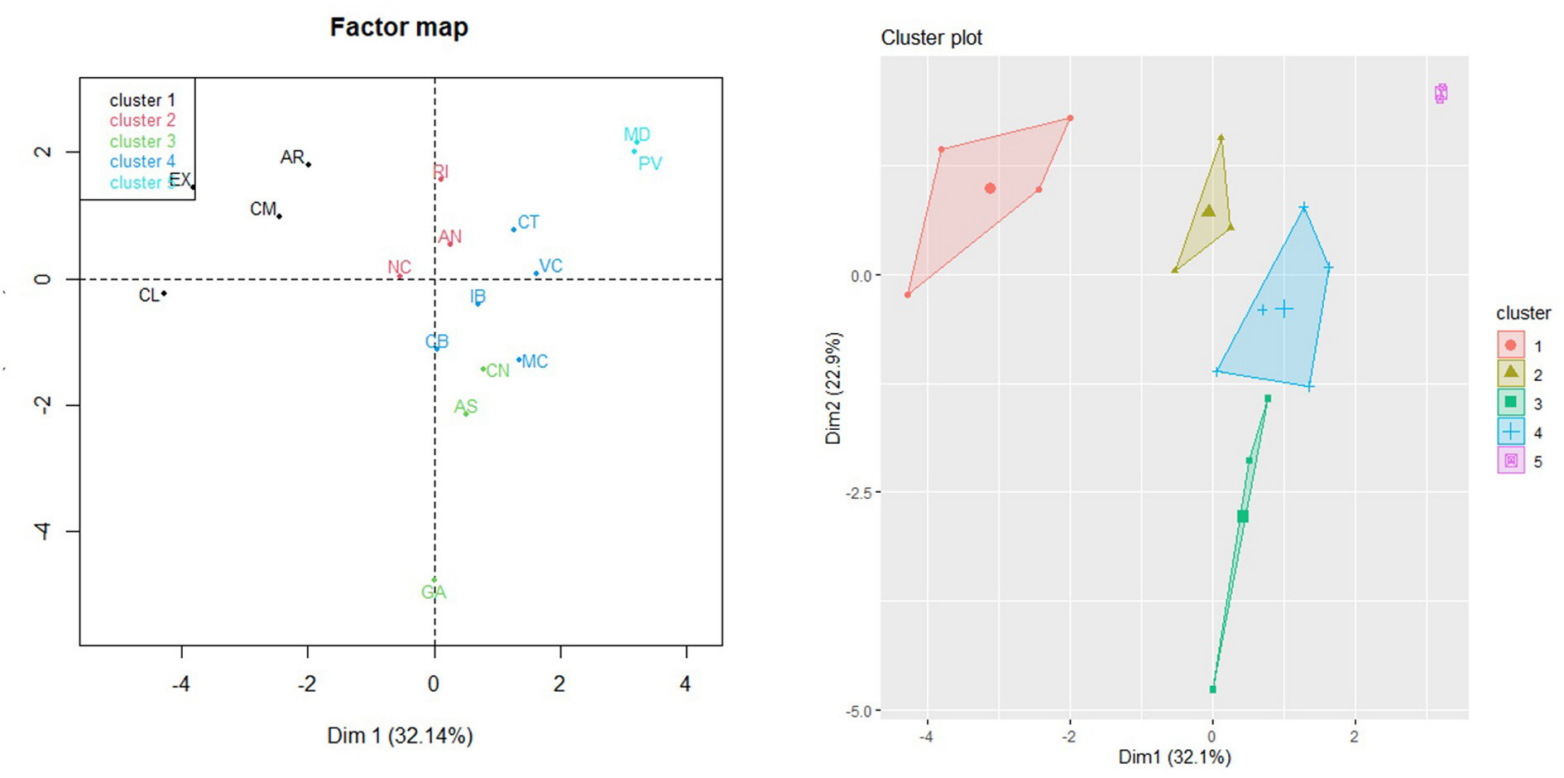
Factor map of ACS. Dimension 1, Dimension 2, and “housing_density.”

Figure 1 represents the distribution of the ACS analyzed in the map factor. In addition, each region has been brought together according to its category in the “housing_density” variable. We can see that the ACS with the lowest density of households per km^2^ are those with the highest percentage of ADSL ≥10 MBps and VDSL coverage technologies. By contrast, those with a higher density of households are the ones with the greatest percentage of FTTH broadband technology coverage and connection speeds above 100 Mbps.

**Figure 2:**
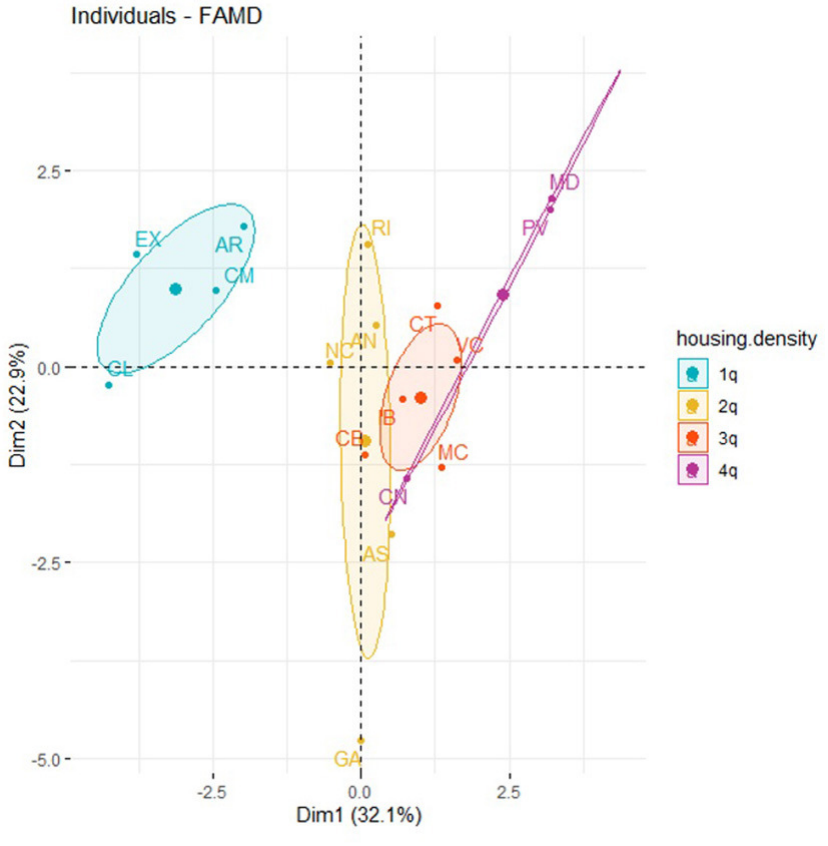
Factor map of ACS in Dimension 1, Dimension 3, and “housing_density.”

**Figure 3:**
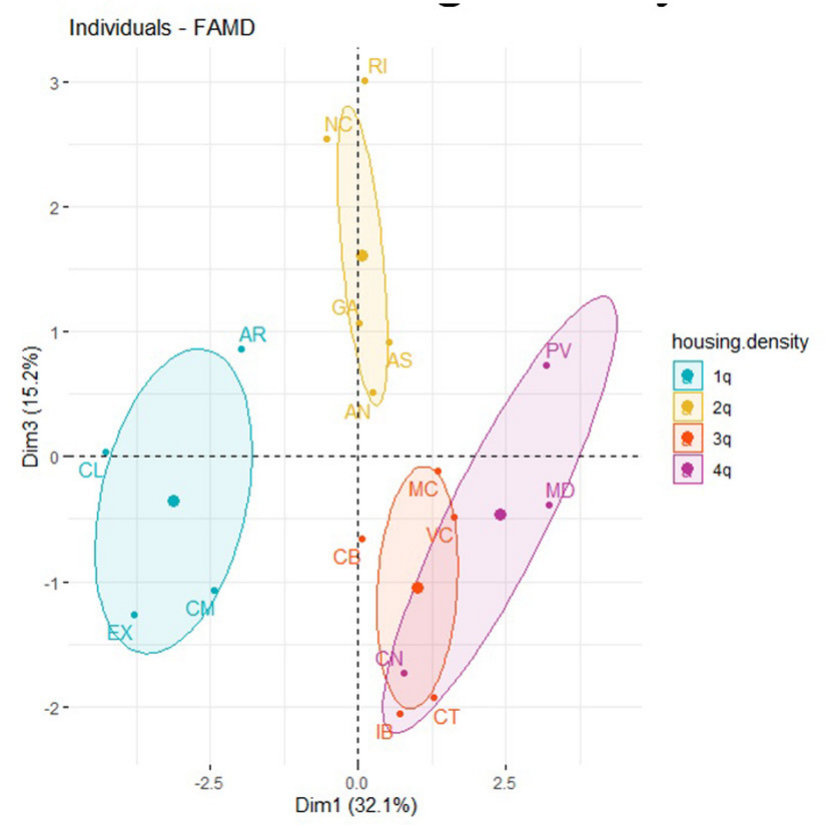
ACS clustering: Five clusters identified by FAMD.

Supplementary Figure S8 (see Appendix 2) and Figures 2, 3 represents the distribution of the ACS according to Dimension 1 (LTE coverage technologies with connection speeds higher than 100 Mbps) and Dimension 3 (LTE broadband coverage and connection speeds higher than 30 Mbps).

Finally, we obtained the grouping of the ACS into five clusters (Figure 1) using FAMD.

- **Cluster 1** (c1) groups the following ACS: Castilla y León (CL), Castilla La Mancha (CL), Aragón (AR), and Extremadura (EX).
- **Cluster 2** (c2) groups the following regions: La Rioja (RI), Andalucía (AN), and Navarra (NC).
- **Cluster 3** (c3) groups the following regions: Galicia (GA), Asturias (AS), and the Canary Islands (CN).
- **Cluster 4** (c4) groups the following regions: Cantabria (CB), the Balearic Islands (IB), Murcia (MC), Catalonia (CT), and Valencia.
- **Cluster 5** (c5) groups the following regions: Madrid (MD) and Basque Country (PV).

**Figure 4:**
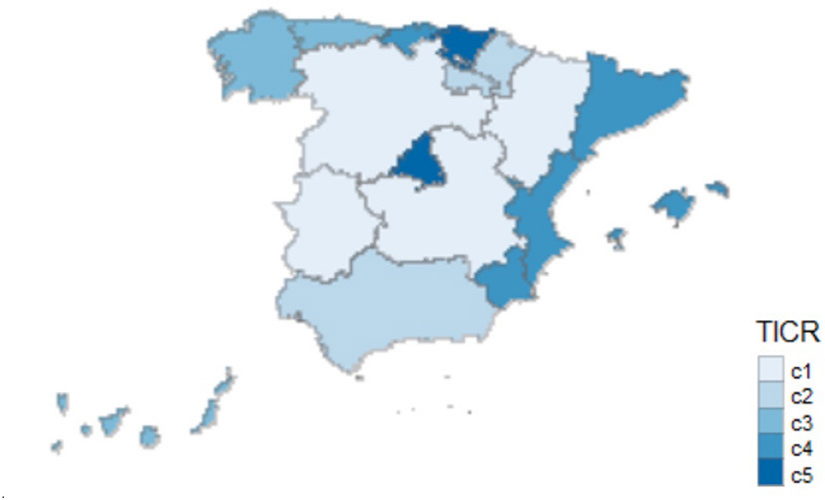
Clusters of the ACS according to the TICR variable.

Appendix 1 and Figure 4 plots, geographically, the clusters of the ACS according to variables “TICR”, “amputation”, “hospital”, “healthcare”, “broadband”, “narrowband”, “education”, “hits.dm”, “health.info.pop”, “povertry”, “hardware.cost” and “connect.cost”. Boxplots of each of these variables are brought together in Appendix 3. We used TICR as categorical variable in the second phase of this research jointly with variables related to DM, SDOH for all the ACS in Spain.

Table 4 summarizes the descriptive statistics of the variables related to DM, such as the number of hospitalizations (“hospital”) and amputations (“amputation”). On the other hand, the database has been filled in with variables related to the SDOH, such as education level and poverty. In addition, an analysis of the technological determinants of these regions was needed, such as the type of connection (broadband or narrowband) and the economic reasons for not having internet access at home, either due to the cost of the computer equipment to connect (“hardware.cost”) or the connection cost (“connet.cost”). Degree of broadband coverage for each type of technology and its connection speed (TICR) has been analyzed. It also included the use of the internet (aggregated for the ACS as a whole) for searches on health-related information in general (“health.info.pop”), and the volume of online searches for the keyword “diabetes mellitus” (“hits.dm”). Finally, we included the overall health spending per patient managed by each ACS.

**Table 4:** Summary of the DM variables, determinants of health, health information searches, type of connection, and reasons for non–use of the internet at home.

	**Education**	**Poverty**	**Hits.dm**	**Health.info.pop**	**Hospital**	**Amputation**	**Broadband**	**Narrowband**	**Hardware.cost**	**Connect.cost**	**Healthcare**
Mean	38,58	19,33	52,18	59,96	6,14	0,08	99,65	1,56	27,70	26,33	1633,70
Variance	61,77	58,46	131,63	12,64	1,63	0,00	0,07	0,54	68,56	61,71	19008,96
Standard deviation	7,86	7,65	11,47	3,56	1,28	0,03	0,26	0,73	8,28	7,86	137,87
Skewness	0,19	0,26	−0,11	−0,17	1,33	0,77	−0,33	0,30	1,23	1,04	−0,89
Kurtosis	2,36	1,77	2,64	2,66	5,70	5,14	1,51	2,50	5,44	5,74	3,91
Median	38,60	17,90	52,44	60,50	5,98	0,08	99,70	1,50	26,60	25,70	1621,15
Minimun	25,90	7,70	31,31	53,40	4,59	0,03	99,20	0,50	16,40	13,70	1321,14
Maximun	53,70	31,50	72,90	66,10	9,70	0,16	100,00	3,00	50,50	47,60	1851,86
Range	27,80	23,80	41,60	12,70	5,11	0,13	0,80	2,50	34,10	33,90	530,72
Count	17,00	17,00	17,00	17,00	17,00	17,00	17,00	17,00	17,00	17,00	17,00
1st cuartil	33,10	12,90	44,77	57,80	5,13	0,07	99,40	0,90	21,10	22,90	1572,82
3rd cuartil	42,70	26,20	58,04	61,70	6,61	0,10	99,90	1,90	29,50	27,90	1735,43
Interquartile range	9,60	13,30	13,27	3,90	1,48	0,03	0,50	1,00	8,40	5,00	162,61

Source: Ministry of economic affairs and digital transformation (state secretariat for digitalization and artificial intelligence department); instituto nacional de estadística; ministry of health.

Table 5 outlines the correlations between the abovementioned variables.

**Table 5:** Correlations between variables “education”, “poverty”, “hits.dm”, “health.info.pop”, “hospital”, “amputation”, “broadband”, “narrowband”, “hardware.cost”, “connect.cost”, “healthcare”.

	**Education**	**Poverty**	**Hits.dm**	**Health.info.pop**	**Hospital**	**Amputation**	**Broadband**	**Narrowband**	**Hardware.cost**	**Connect.cost**	**Healthcare**
Education	1,00	0,64	0,25	−0,32	0,08	0,52	−0,20	0,34	0,46	0,46	−0,12
Poverty	0,64	1,00	0,31	−0,02	−0,22	0,75	−0,09	0,16	0,51	0,53	−0,26
Hits.dm	0,25	0,31	1,00	−0,16	−0,49	0,27	0,36	−0,08	0,10	0,08	−0,39
Health.info.pop	−0,32	−0,02	−0,16	1,00	−0,15	−0,25	0,11	−0,44	0,20	0,14	−0,14
Hospital	0,08	−0,22	−0,49	−0,15	1,00	−0,10	−0,27	−0,05	0,32	0,43	0,20
Amputation	0,52	0,75	0,27	−0,25	−0,10	1,00	−0,37	0,41	0,44	0,45	−0,04
Broadband	−0,20	−0,09	0,36	0,11	−0,27	−0,37	1,00	−0,63	−0,04	−0,16	0,10
Narrowband	0,34	0,16	−0,08	−0,44	−0,05	0,41	−0,63	1,00	−0,12	−0,05	0,23
Hardware.cost	0,46	0,51	0,10	0,20	0,32	0,44	−0,04	−0,12	1,00	0,91	−0,17
Connect.cost	0,46	0,53	0,08	0,14	0,43	0,45	−0,16	−0,05	0,91	1,00	−0,18
Healthcare	−0,12	−0,26	−0,39	−0,14	0,20	−0,04	0,10	0,23	−0,17	−0,18	1,00

Source: Ministry of economic affairs and digital transformation (state secretariat for digitalization and artificial intelligence department); instituto nacional de estadística; ministry of health.

**Figure 5:**
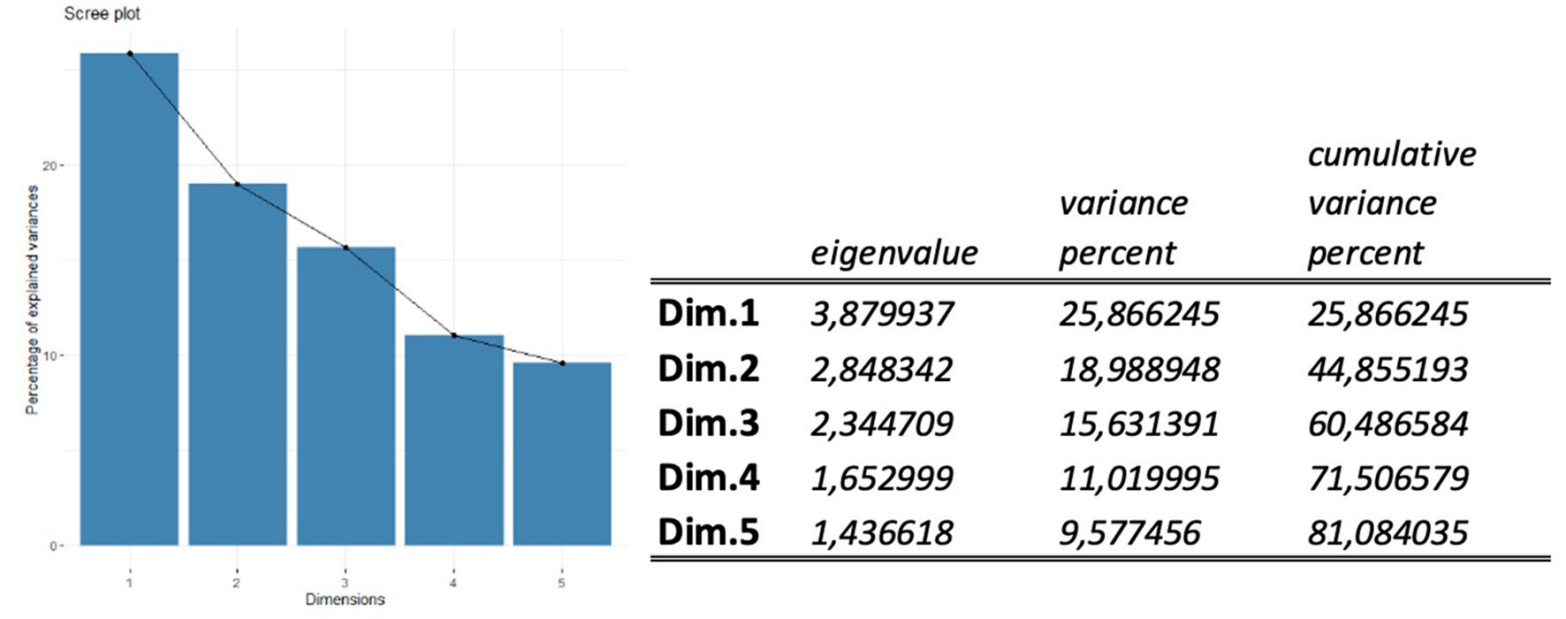
Percentage of inertia explained by each FAMD dimensions. SDOH and DM variables.

In reference to Figure 5, the first two dimensions explain 44.85% of the cumulative variance percent. Dimension 1 (Table 6 and Figure 5) explains 25.86% of the variance, clustering the variables of “education,” “poverty,” “amputation,” and “connect.cost.” Dimension 1 (Supplementary Figure S9, see Appendix 2) summarizes (by its level of contribution to this dimension) the number of amputations caused by DM, along with the socioeconomic factors linked to the level of education and poverty as SDOH, as well as the economic reasons given for not having an internet connection at home (Supplementary Figures S9–S14, see Appendix 2).

**Table 6:** Contributions to the dimensions of DM and the SDOH variables.

	**Dim.1**	**Dim.2**	**Dim.3**	**Dim.4**	**Dim.5**
Education	16,685	0,075	0,250	4,207	5,882
Poverty	16,793	1,420	2,967	2,154	0,096
Hits.dm	0,945	4,455	24,115	0,022	6,518
Health.info.pop	1,839	6,805	2,098	16,147	1,782
Hospital	0,309	0,061	28,526	9,671	0,429
Amputation	18,450	0,292	1,901	2,542	5,099
Broadband	4,843	13,949	8,373	7,302	1,073
Narrowband	5,194	21,400	0,506	0,161	0,007
Hardware.cost	10,688	13,172	3,270	0,031	2,212
Connect.cost	12,594	8,804	5,332	0,024	0,112
Healthcare	0,441	3,670	0,288	20,477	26,868
TICR	11,220	25,897	22,375	37,261	49,921

Dimension 2 explains 18.98% of the variance percent (Supplementary Figure S10, see Appendix 2) clustering the variables “narrowband,” “hardware.cost,” and “broadband” related to the types of internet connection speed in people's homes and the economic reasons for not using the internet at home in relation to the cost of computer equipment.

Dimension 3 explains 15.63% of the variance percent (Supplementary Figure S11, see Appendix 2) shows the variables related to the volume of online searches for the term “diabetes mellitus” (RSV), together with the number of hospitalizations caused by DM. Dimension 4 (Supplementary Figure S12, see Appendix 2) represents the variable “health.info.pop,” which relates to people's use of the internet to search for information on health-related topics in general.

Lastly, Dimension 5 includes the variables of healthcare spending per patient managed by the ACS (healthcare), and the variable that categorizes each region analyzed according to its level of broadband coverage by technology and connection speed (TICR). See Supplementary Figure S13 (Appendix 2).

**Figure 6:**
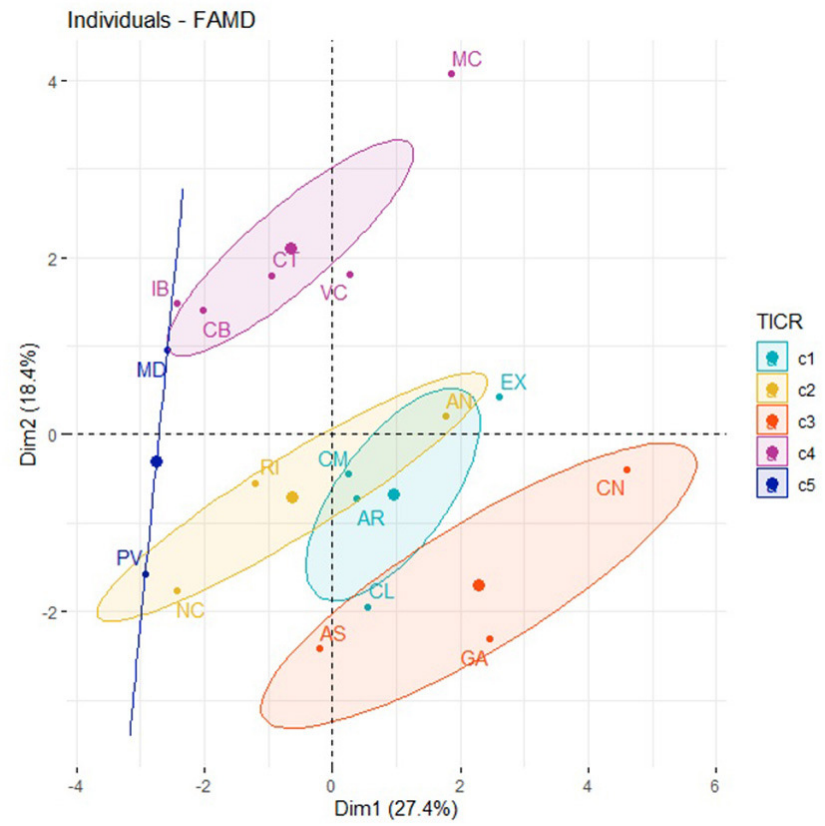
Factor map of the ACS, Dimension 1, Dimension 2, and TICR.

Figure 6 represents the distribution of the ACS in the map factor and in which, in turn, each region is categorized according to the value obtained in the TICR variable. Supplementary Figure S14 (see Appendix 2) plots the circle of correlations of the quantitative variables, which allowed us to check the clusters between variables, as well as their level of contribution to the corresponding dimensions.

Thus, the results obtained previously in the correlation coefficients in Table 5 show these variables related to amputations as a consequence of DM (“amputation”), together with SDOH such as poverty, education level (“education”), and internet connection cost (“connect.cost”). Figure 6 represents ACS with the highest level of amputations caused by DM, with regions that in turn have socioeconomic conditions of greater poverty, lower levels of education, and a higher share of the population whose reason for not having internet access for individual use derives from economic reasons due to the cost of the connection. Regarding Dimension 2 (Supplementary Figure S14, see Appendix 2) and as mentioned before (according to Table 5), the variables related to the proportion of the population that has a narrowband vs. broadband internet connection don't have internet at home due to the economic cost of computer equipment.

In Figure 6, it's highlighted that the existence of a greater number of amputations caused by DM, coincides with more severe socioeconomic conditions (poverty and education level). A greater amount of the population is unable to have an internet connection and computer equipment for economic reasons. It should be noted that there is a coincidence of higher numbers of amputations, higher poverty, and lower education level. We also identified a higher share of the population that is using narrowband for an internet connection. In other words, the areas with the lowest levels of broadband coverage and connection speed (coinciding with the highest share of the population reporting the use of narrowband for an internet connection) are also areas where there are higher poverty rates and lower education levels and, in turn, more economic difficulties in accessing the internet. However, these regions are also characterized, compared to the rest, by higher numbers of amputations due to DM.

Figure 6 also shows ACS with lower amputation rates, but also with better socioeconomic conditions (poverty and education), together with lower rates of narrowband housing, fewer cases of not using the internet due to economic cost, and greater use of broadband. In addition, there is a coincidence of the ACS which, according to the TICR classification, belongs to clusters 2, 4 and 5. This implies that a greater percentage of the population has a broadband connection and speeds higher than 100 Mbps.

**Figure 7:**
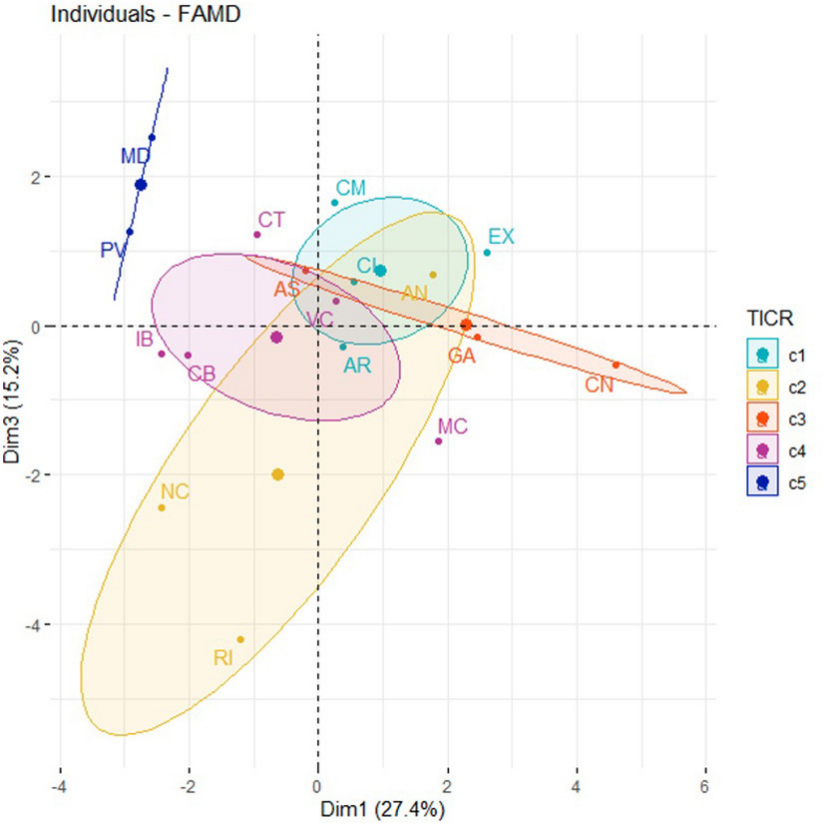
Factor map of the ACS. Dimension 1, Dimension 3, and TICR.

Next, we analyzed the relationship between the variables linked to DM (“hospital” and “amputation”), socioeconomic factors (“poverty” and “education”), types of internet connection speeds (“broadband” and “narrowband”), and the share of the population that uses the internet to search for information about health in general (“health.info.pop”) and DM in particular (“hits.dm”). According to Supplementary Figure S15 (see Appendix 2), there is no correlation of the hits.dm variable with socioeconomic factors, type of internet connection band, economic reasons for not using the internet, or the amputation rate. If we compare this relationship in Supplementary Figure S16 (see Appendix 2), we can also observe that the variable health.info.pop is not related to the health determinant variable (the amputation rate), to the type of connection, or to the reasons for using or not using the internet. However, as with the “hits.dm,” the variable for searching for information on the internet on health issues (“health.info.pop”) also has a negative relationship with the rate of hospitalizations caused by DM (“hospital”), although less than the variable referred to RSV on “hits.dm.” Figure 7, Supplementary Figure S17 shows that there isn't a TICR pattern in ACS (Supplementary Figures S16–S18, see Appendix 2).

**Figure 8:**
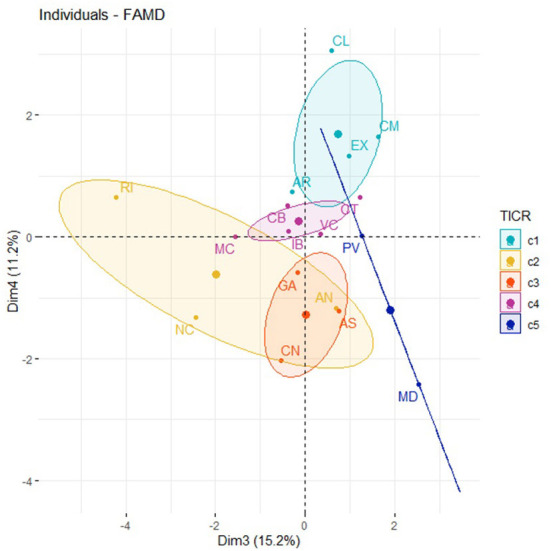
Factor map of the ACS, Dimension 3, Dimension 4, and the TICR.

Dimensions 3 and 4 (Supplementary Figure S18, see Appendix 2), variable “health.info.pop” is not correlated with “hits.dm” or with “hospital.” There isn't a TICR pattern in ACS in Figure 8.

**Figure 9:**
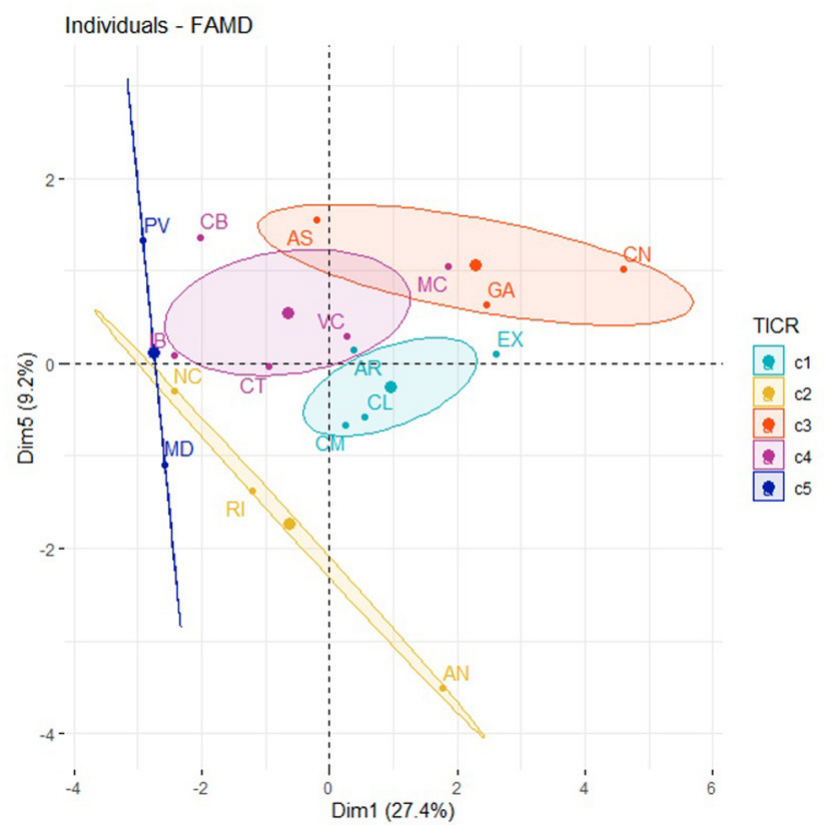
Factor map of the ACS, Dimension 1, Dimension 5, and the TICR.

Finally, we explored the distribution of the ACS according to dimensions 1 and 5. Based on the clusters obtained according to the correlations between the variables (Supplementary Figure S19, see Appendix 2), healthcare spending per patient managed by the ACS (“healthcare”) is not correlated with amputation rate (“amputation”) or with the social and economic conditioning factors of the regions (“poverty” and “education”), or with the economic reasons for not having an internet connection for individual use (“hardware.cost” and “connect.cost”). Figure 9 shows that there isn't a TICR pattern in ACS.

## Discussion

The present study coincides with the conclusions of prior research, discussed above, related to inequality and the use of ICT (5, 17–19, 54). Likewise, we also agree with (53) on the importance of education and inequality in internet use, and with (10) approach to internet use. We agree with (2) on what kinds of inequalities can affect society.

Our results revealed a greater number of amputations in regions (ACS) with a higher rate of poverty and a lower level of education. These are regions with the highest number of individuals who have declared, according to official statistics, that they do not use the internet for economic reasons, especially because of connection costs. Among those who do use it, they state that the connections have been through narrowband. Furthermore, according to the variable obtained in this study, these ACS are regions with the greatest percentage of internet coverage among the set of technologies analyzed and at the lowest speed compared to the other regions.

On the other hand, ACS with fewer amputations have lower aggregate poverty rates, higher levels of education, and fewer individuals who report not using the internet for economic reasons. Moreover, with regard to the variable created, TICR, these are regions with greater internet coverage on average in comparison to the rest of the ACS among the set of technologies, and with higher connection speeds. Regarding the search for online information on health issues in general and DM in particular, we noted that people in ACS with a lower number of hospitalizations had a greater interest in searching for the term “diabetes mellitus,” and, unexpectedly, in regions with a higher number of hospitalizations due to DM. We did not detect any relationship between hospitalizations due to DM and use of the internet declared by users regarding searches on topics about health in general. In relation to online searches and internet coverage and connection speeds, the results were disparate, and we observed no pattern between the regions. This was the case with health care costs per patient and the variable TICR, with different relationships between the ACS.

This study on the effects of DM in Spain, related to the rates of amputations and hospitalizations as a consequence of DM, includes as a novelty the use of technological determinants of health. To this end, and as a contribution of this work, we obtained the TICR variable in the initial phase using FAMD. This variable classifies the ACS according to their percentage of broadband coverage based on different existing technologies, as well as connection speeds. Subsequently, this new qualitative variable that categorizes the Spanish regions according to their ICT infrastructure has been linked to variables related to the type of internet connection, the reason for using the internet to search for information on health in general and DM in particular, and economic reasons for not having internet access at home. This set of technological determinants of health has been tied to health-related socioeconomic variables (poverty and education level), in addition to health spending.

Please note that, as a limitation of this study, we extracted the sources (aggregated data) of the database from official institutions. Ceuta and Melilla (two Spanish ACS) were excluded from this study because some variables needed were not available. Furthermore, we extracted the data related to the monitoring of interest in DM through the internet *via* GT; this tool is linked to user searches on the Google search engine. Please, be aware that in this research, the results were not classified by population age ranges in patients with DM due to the technological variables used in this study. Data available did not show these demographic characteristics. In addition, the multivariate methodology applied has been useful for measuring and explaining the degree of relationships between the selected variables.

This research is propitious to open new lines of work, such as performing an exhaustive study of the same in each autonomous community, adding more variables related to the telecommunications sector. It would also be interesting to evaluate more sequelae resulting from DM and, for this purpose, to use microdata. The information generated would be useful for public policymakers and, in this way, helpful for generating proposals for citizens to promote the use of ICT, education, and ultimately contribute to the development of eHealth.

## Data availability statement

Publicly available datasets were analyzed in this study. This data can be found here: Spain's Health Ministry, http://inclasns.msssi.es/main.html; Ministry of Economic Affairs and Digital Transformation, https://portal.mineco.gob.es/; Google Trends, https://trends.google.es; Spain's National Institute of Statistics, https://www.ine.es.

## Author contributions

Conceptualization, formal analysis, investigation, and writing—original draft preparation: IBF. Methodology, resources, and data curation: IBF and FCV. Validation and supervision: IBF, FCV, MCL, and MJPL. Writing—review and editing: IBF, FCV, and MCL. Project administration: MCL and IBF. All authors have read and agreed to the published version of the manuscript.
