# Supplementary material for: Diabetes mellitus and inequalities in the equipment and use of information technologies as a socioeconomic determinant of health in Spain
Source: Front Public Health. 2023 Jan 9;10:1033461. doi: 10.3389/fpubh.2022.1033461 (PMC9868750; doi:10.3389/fpubh.2022.1033461)
Supplement: Supplementary file 1 [file Data_Sheet_1.pdf]

Appendix 1

Maps of Spain

Clusters of the ACS according to variables “amputation”, “hospital”, “healthcare”, “broadband”, “narrowband”, “education”, “hits.dm”, “health.info.pop”, “poverty”, “hardware.cost” and “connect.cost”.

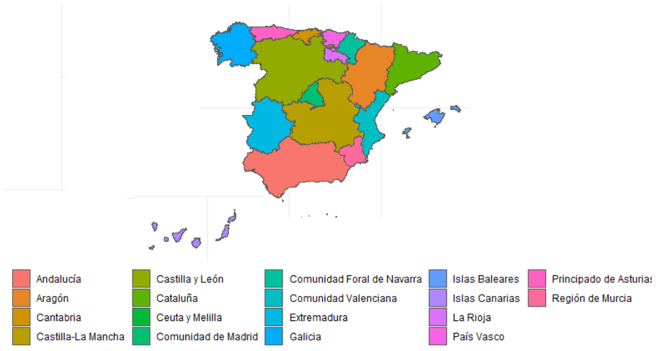

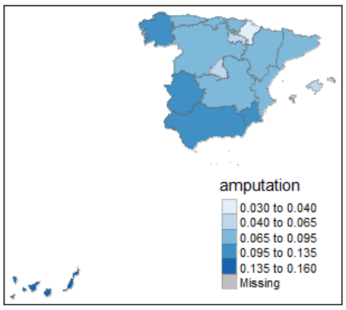

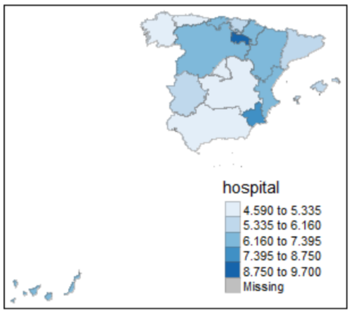

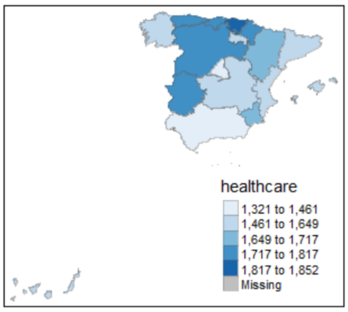

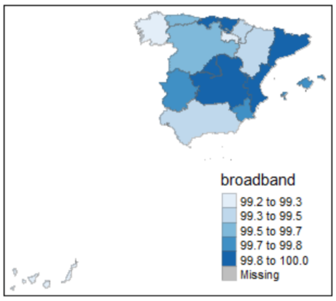

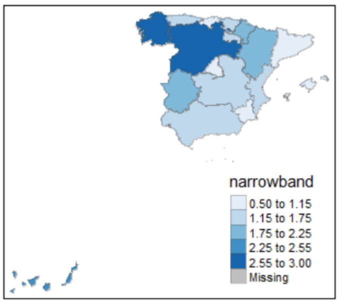

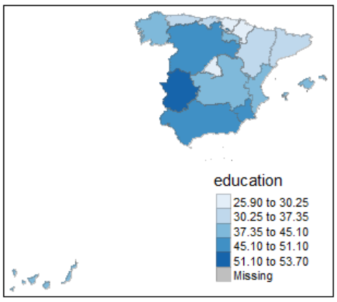

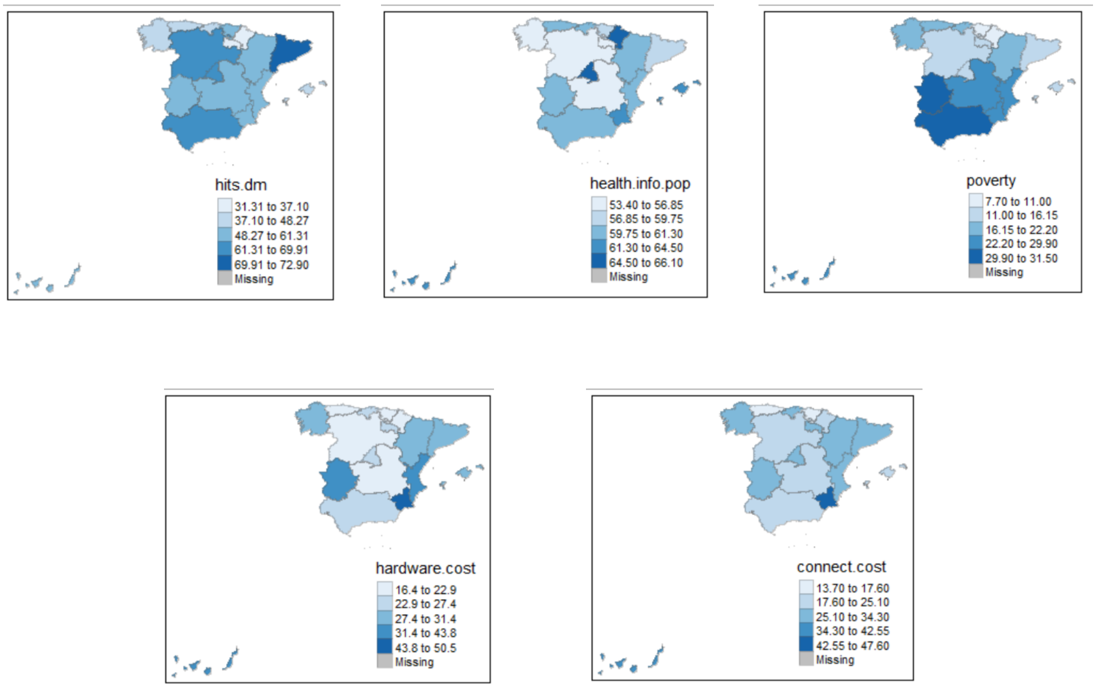

Source: author's own elaboration (data retrieved from Spain’s Health Ministry)
