# Supplementary material for: Diabetes mellitus and inequalities in the equipment and use of information technologies as a socioeconomic determinant of health in Spain
Source: Front Public Health. 2023 Jan 9;10:1033461. doi: 10.3389/fpubh.2022.1033461 (PMC9868750; doi:10.3389/fpubh.2022.1033461)
Supplement: Supplementary file 2 [file Data_Sheet_2.pdf]

APPENDIX 2

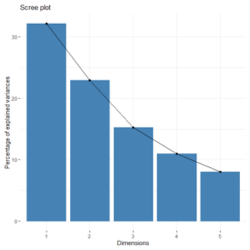

Figure 1 (S1): Screen Plot. Percentage of inertia explained by each FAMD dimensions

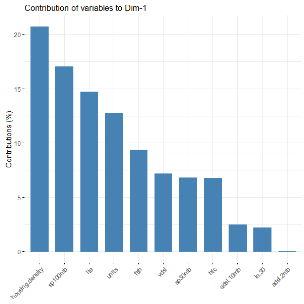

Figure 2 (S2): Contribution of the variables to Dimension 1

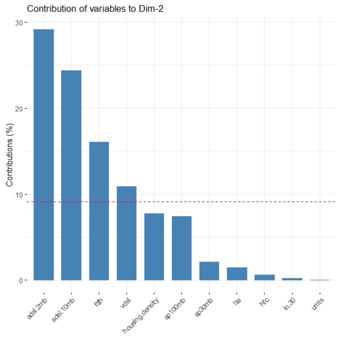

Figure 3 (S3): Contribution of the variables to Dimension 2

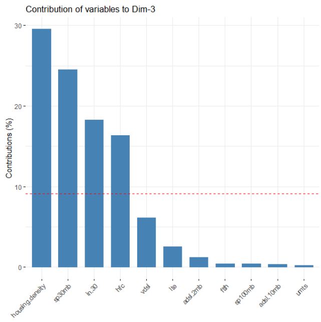

Figure 4 (S4): Contribution of the variables to Dimension 3

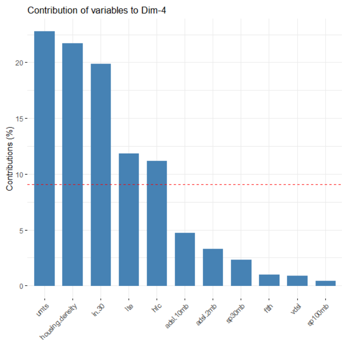

Figure 5 (S5): Contribution of the variables to Dimension 4

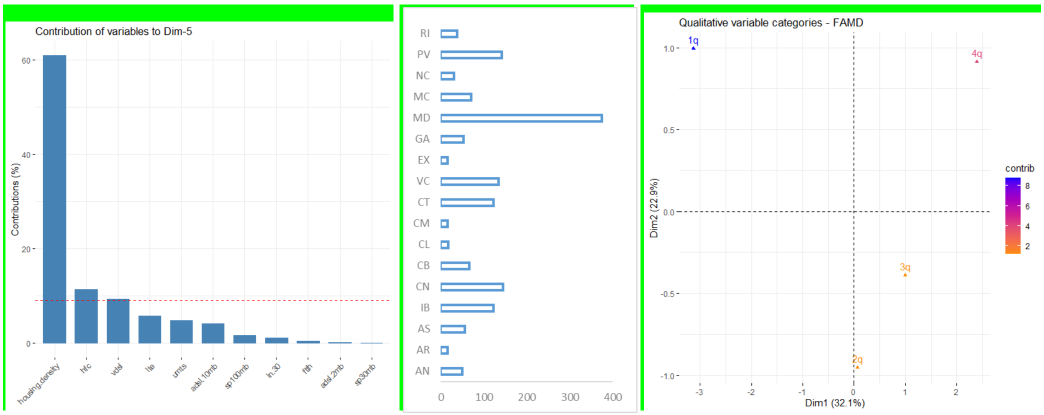

Figure 6 (S6): Contribution of the variables to Dimension 5. Distribution of housing density. Factor map of the qualitative variable “housing_density.”

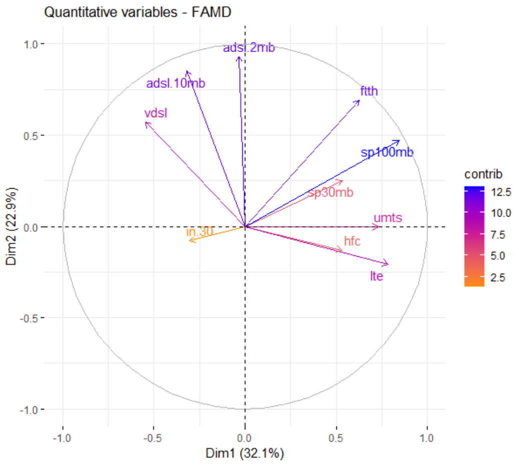

Figure 7 (S7): Correlation circle shows the relationship between quantitative variables and their contribution to dimensions 1 and 2.

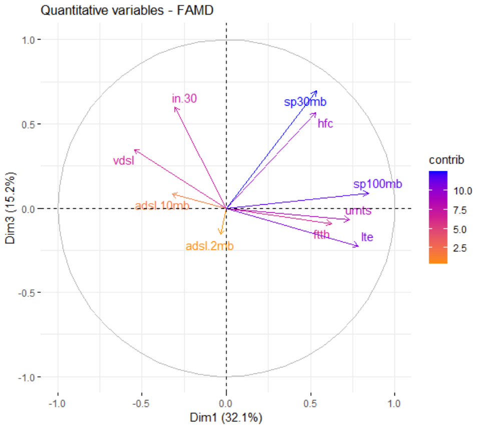

Figure 8 (S8): Correlation circle shows the relationship between quantitative variables and their contribution to Dimensions 1 and Dimension 3.

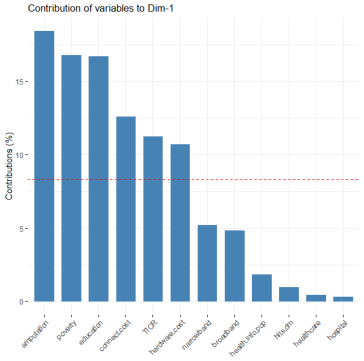

Figure 9 (S9): Contribution of the variables to Dimension 1

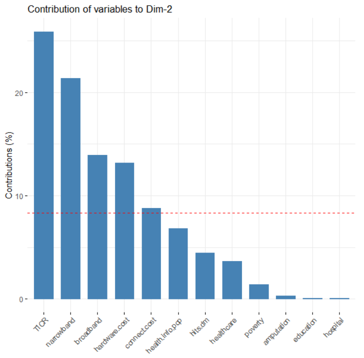

Figure 10 (S10): Contribution of the variables to Dimension 2

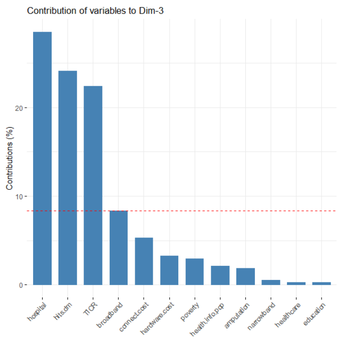

Figure 11 (S11): Contribution of the variables to Dimension 3

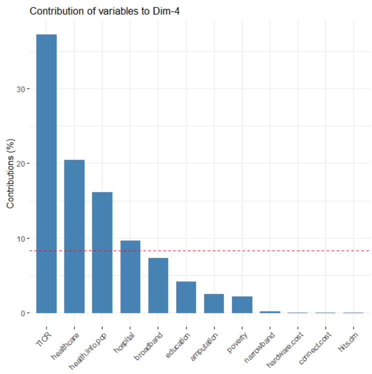

Figure 12 (S12): Contribution of the variables to Dimension 4

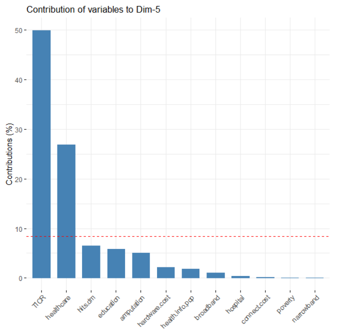

Figure 13 (S13): Contribution of the variables to Dimension 5

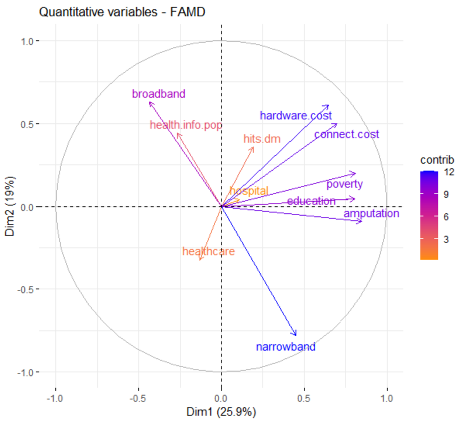

Figure 14 (S14): Correlation circle, Dimension 1 and Dimension 2.

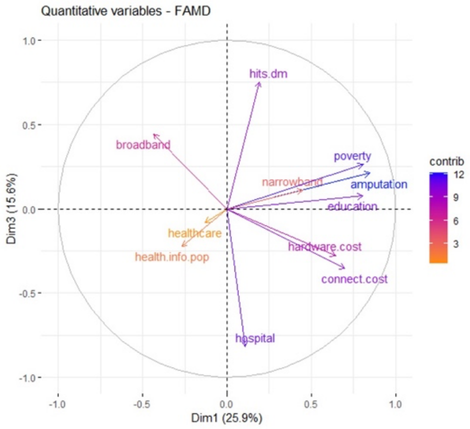

Figure 15 (S15): Correlation circle, Dimension 1 and Dimension 3.

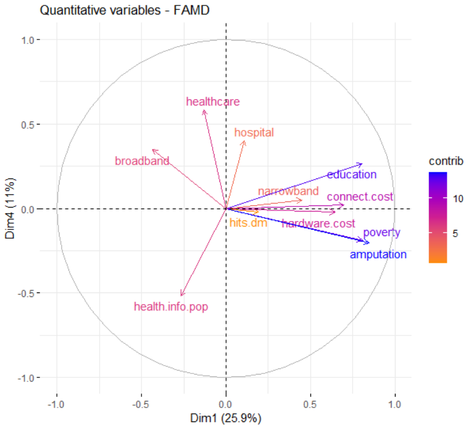

Figure 16 (S16): Correlation circle, Dimension 1 and Dimension 4.

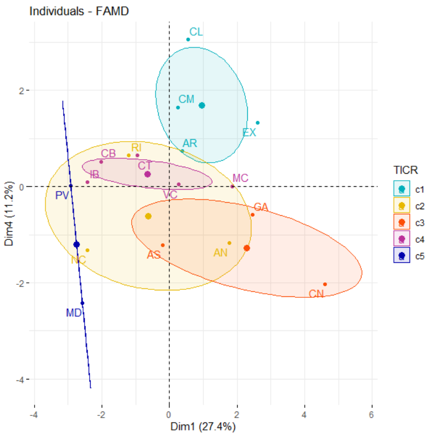

Figure 17 (S17): Factor map of the ACS, Dimension 1, Dimension 4, and the TICR.

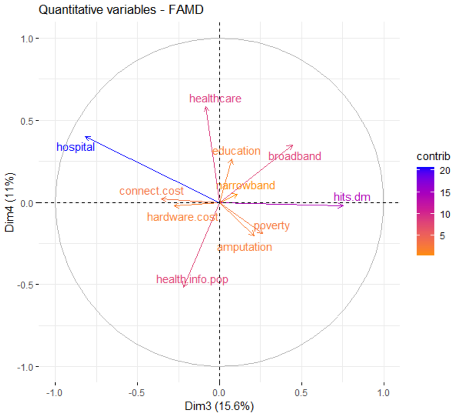

Figure 18 (S18): Correlation circle, Dimension 3 and Dimension 4.

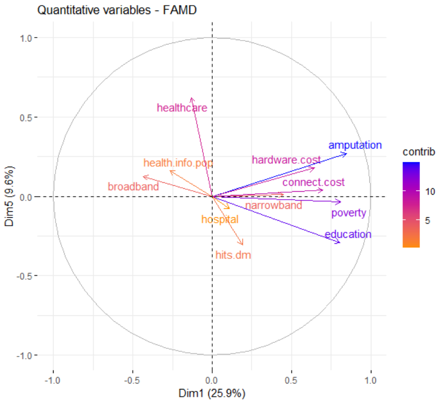

Figure 19 (S19): Correlation circle, Dimension 1 and Dimension 5.
