# Supplementary figures and images for: Diabetes mellitus and inequalities in the equipment and use of information technologies as a socioeconomic determinant of health in Spain
Source: Front Public Health. 2023 Jan 9;10:1033461. doi: 10.3389/fpubh.2022.1033461 (PMC9868750; doi:10.3389/fpubh.2022.1033461)

## APPENDIX 3

### Boxplots

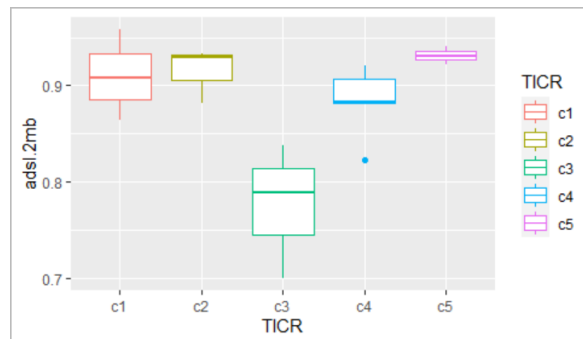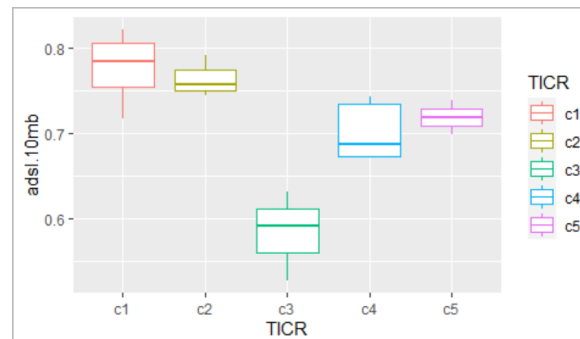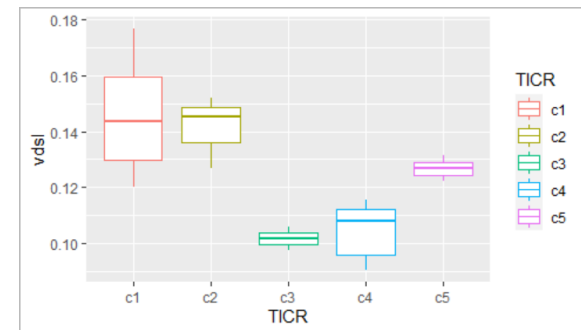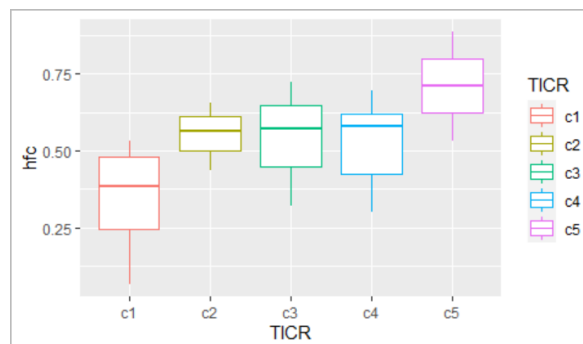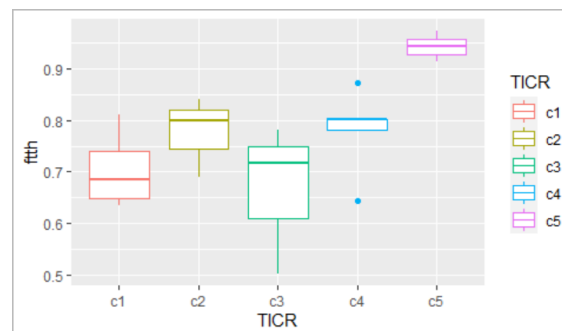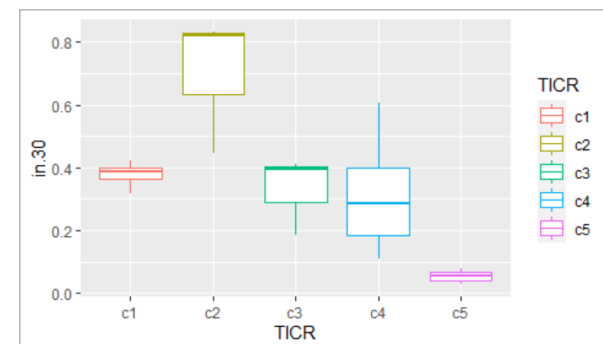

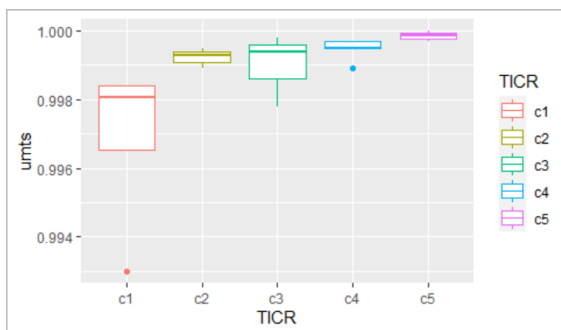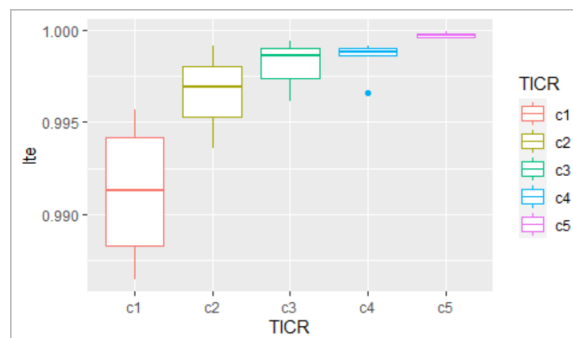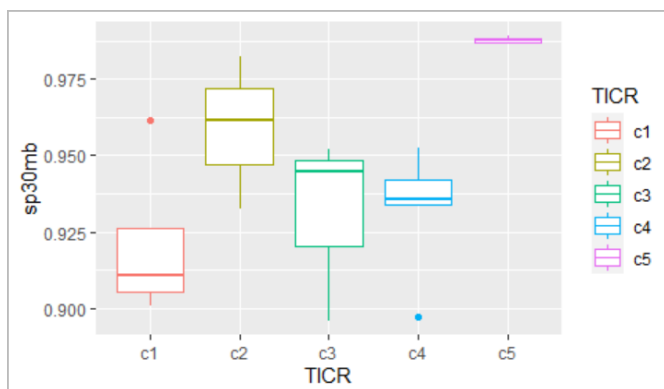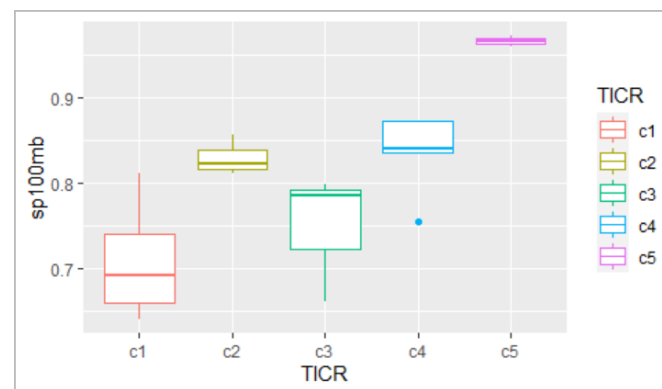

Supplement: Supplementary file 3 [file Data_Sheet_3.pdf]
